# Supplementary material for: Racial Disparities in Periprosthetic Joint Infections after Primary Total Joint Arthroplasty: A Retrospective Study
Source: Antibiotics (Basel). 2023 Nov 16;12(11):1629. doi: 10.3390/antibiotics12111629 (PMC10668943; doi:10.3390/antibiotics12111629)
Supplement: Supplementary file 1 [file antibiotics-12-01629-s001.zip › antibiotics-2664224-supplementary.pdf]

antibiotics MDPI

Supplementary Material

Table S1. Current Procedural Terminology (CPT®) and International Classification of Diseases, Tenth Revision (ICD-10) codes.

	Procedure or Diagnosis
CPT codes	
27130	Primary total hip arthroplasty
27447	Primary total knee arthroplasty
ICD-10 codes	
T84.5	Infection and inflammatory reaction due to internal joint prosthesis
T84.51	Infection and inflammatory reaction due to internal right hip prosthesis
T84.52	Infection and inflammatory reaction due to internal left hip prosthesis
T84.53	Infection and inflammatory reaction due to internal right knee prosthesis
T84.54	Infection and inflammatory reaction due to internal left knee prosthesis

Table S2. Baseline characteristics by race and sex intersection groups.

Characteristic	NH White Male (N = 4562) no. (%)	NH White Female (N = 5102) no. (%)	NH Black Male (N = 128) no. (%)	NH Black Female (N = 226) no. (%)
Age – years, mean (±SD)	68 (±10)	70 (±10)	62 (±11)	67 (±11)
Median Household Income† – mean (±SD)	108,891 (±39,776)	106,843 (±39,001)	83,687 (±31,619)	82,193 (±31,883)
Type of TJA*				
TKA*	2363 (51.8)	2852 (55.9)	58 (45.3)	135 (59.7)
THA*	2199 (48.2)	2250 (44.1)	70 (54.7)	91 (40.3)
Comorbidities				
Obesity	1253 (27.9)	1541 (30.2)	42 (32.8)	126 (55.8)
Hypertension, uncomplicated	2978 (65.3)	3088 (60.5)	95 (74.2)	184 (81.4)
Hypertension, complicated	594 (13.0)	559 (11.0)	26 (20.3)	59 (26.1)
Chronic pulmonary disease	903 (19.8)	1352 (26.5)	33 (25.8)	87 (38.5)
Diabetes, uncomplicated	718 (15.7)	613 (12.0)	36 (28.1)	78 (34.5)
Diabetes, complicated	482 (10.6)	372 (7.3)	27 (21.1)	46 (20.4)
Renal failure	531 (11.6)	509 (10.9)	27 (21.1)	31 (13.7)
Liver disease	474 (10.4)	541 (10.6)	21 (16.4)	38 (16.8)
HIV/AIDS	17 (0.4)	4 (0.1)	2 (1.6)	1 (0.4)
Metastatic cancer	260 (5.7)	280 (5.5)	4 (3.1)	9 (4.0)
Rheumatological disorders	456 (10.0)	805 (15.8)	13 (10.2)	57 (25.2)
Blood loss anemia	116 (2.5)	156 (3.1)	8 (6.3)	18 (8.0)
Deficiency anemia	351 (7.7)	480 (9.4)	12 (9.4)	49 (21.7)
Alcohol abuse	322 (7.1)	184 (3.6)	13 (10.2)	11 (4.9)
Drug abuse	322 (7.1)	263 (5.2)	22 (17.2)	22 (9.7)
Psychoses	28 (0.6)	53 (1.0)	3 (2.3)	5 (2.2)
Depression	882 (19.3)	1,700 (33.3)	29 (22.7)	89 (39.4)
ECI* – mean (±SD)	18.2 (±23.6)	18.3 (±21.6)	25.9 (±28.3)	28.4 (±26.1)

*Abbreviations: NH—non-Hispanic; TJA—Total Joint Arthroplasty; THA—Total Hip Arthroplasty; TKA—Total Knee Arthroplasty; ECI—Elixhauser Comorbidity Index. † Median household income was calculated using Zip Code data.

Table S3. Bivariate associations between variables and PJI.

Variable	PJI	
	**Cumulative Incidence Ratio (95% CI)**	***p*-value**
Type of Arthroplasty		<0.0001
Hip Arthroplasty	Ref	
Knee Arthroplasty	17.26 (8.50–35.07)	
Obesity		0.0092
No obesity	Ref	
Obese	1.51 (1.09–1.08)	
Diabetes		0.0115
No diabetes	Ref	
Diabetes	1.59 (1.11–2.23)	
Substance abuse		<0.0001
No substance abuse	Ref	
Substance abuse	2.45 (1.61–3.74)	

*Abbreviations: PJI—periprosthetic joint infection.

Table S4. Baseline characteristics in individuals with and without PJI, stratified by race.

Characteristic	NH White		NH Black	
	**PJI (N = 159) no. (%)**	**No PJI (N = 9505) no. (%)**	**PJI (N = 11) no. (%)**	**No PJI (N = 343) no. (%)**
Age – years, mean (±SD)	70 (±10)	69 (±10)	67 (±9)	65 (±12)
Sex – no. (%)				
Female	61 (38.4)	5041 (53.0)	6 (54.5)	220 (64.1)
Male	98 (61.6)	4464 (47.0)	5 (45.5)	123 (35.9)
Median Household Income† – mean (±SD)	103,762 (±38,056)	107,877 (±39,400)	85,894 (±42,967)	82,633 (±31,366)
Type of TJA*				
TKA*	151 (95.0)	5064 (53.3)	11 (100.0)	102 (53.1)
THA*	8 (5.0)	4441 (46.7)	0 (100.0)	0 (0.0)
Comorbidities				
Obesity	60 (37.7)	2754 (29.0)	6 (54.5)	162 (47.2)
Hypertension, uncomplicated	117 (73.6)	5949 (62.6)	11 (100.0)	268 (78.1)
Hypertension, complicated	43 (27.0)	1110 (11.7)	3 (27.3)	82 (23.)
Chronic pulmonary disease	54 (34.0)	2201 (23.2)	3 (27.3)	117 (34.1)
Diabetes, uncomplicated	33 (20.8)	1298 (13.7)	3 (27.3)	111 (32.4)
Diabetes, complicated	25 (15.7)	829 (8.7)	2 (18.2)	71 (20.7)
Renal failure	28 (17.6)	1012 (10.6)	4 (36.4)	75 (21.9)
Liver disease	27 (17.0)	988 (10.4)	4 (36.4)	55 (16.0)
HIV/AIDS	0 (0.0)	21 (0.2)	0 (0.0)	3 (0.9)
Metastatic cancer	11 (6.9)	529 (5.6)	2 (18.2)	11 (3.2)
Rheumatological disorders	29 (18.2)	1232 (13.0)	1 (9.1)	69 (20.1)
Blood loss anemia	6 (3.8)	266 (2.8)	2 (18.2)	24 (7.0)
Deficiency anemia	26 (16.4)	805 (8.5)	4 (36.4)	57 (16.6)
Alcohol abuse	18 (11.3)	488 (5.1)	1 (9.1)	23 (6.7)
Drug abuse	21 (13.2)	564 (5.6)	3 (27.3)	41 (12.0)
Psychoses	1 (0.6)	80 (0.8)	1 (9.1)	7 (2.0)
Depression	59 (37.1)	2523 (26.5)	6 (54.5)	112 (32.7)
ECI* – mean (±SD)	32.1 (±29.2)	18.0 (±22.4)	48.3 (±42.6)	26.8 (±26.1)

*Abbreviations: TJA—Total Joint Arthroplasty; THA—Total Hip Arthroplasty; TKA—Total Knee Arthroplasty. † Median household income was calculated using Zip Code data.

Table S5. Comparison of Poisson regression models comparing the incidence of PJI between non-Hispanic White and non-Hispanic Black individuals using non-Hispanic White individuals as the reference group.

Model	Cumulative Incidence Ratio (95% CI)	*p*-value
Model 1: Unadjusted	1.89 (1.04, 3.44)	0.0381
Model 2: Adjusted for age and sex	2.12 (1.16, 3.89)	0.0150
Model 3: Adjusted for age, sex and comorbidities (ECI* score)	1.65 (0.90, 3.03)	0.1036

*Abbreviations: ECI—Elixhauser Comorbidity Index.
